# Should we avoid invasive treatment in cancer patients with pericardial tamponade?

**DOI:** 10.1186/cc13280

**Published:** 2014-03-17

**Authors:** F Galas, J Fukushima, E Osawa, C Park, J Almeida, D Nagaoka, L Hajjar

**Affiliations:** 1 Instituto do Cancer do Estado de São Paulo, Brazil

## Introduction

Patients with cancer now live longer due to advances in oncologic treatment. ICU admission is progressively more frequent in this population due to complications of both disease and therapy. Pericardial effusion leading to tamponade and hemodynamic compromise is a common finding in the advanced stages of disease and results in death if not treated on an urgent basis. However, we do not know midterm outcomes of these patients after pericardial drainage. The aim of this study was to evaluate prospectively cancer patients with pericardial tamponade regarding mortality in 6 months.

## Methods

We evaluated consecutively 105 patients with cardiac tamponade consecutively admitted to the ICU of the Cancer Institute, a reference cancer hospital, during a 3-year period. Baseline characteristics and clinical data were collected prospectively. Patients were followed during a 6-month period.

## Results

Fifty-three patients (50%) were female. Most patients had a previous diagnosis of lung neoplasia (46%), followed by breast neoplasia (15%) and hematological neoplasia (15%). The mean Karnofsky performance status of patients was 70. All these patients underwent surgical drainage in a mean time of 1.5 hours since ICU admission until surgery. Length of ICU stay was 8 days (2 to 15) and of hospital stay was 16 days (8 to 31). ICU mortality was 75.2%, hospital mortality was 83% and at 3 months 92% of patients were dead.

## Conclusion

Cardiac tamponade is a serious complication in cancer patients. Despite adequate treatment, high rates of mortality are observed. Prospective studies are needed to better define whether in these patients end-of-life discussion should be implemented early in the diagnosis, avoiding futility.

